# Adipose Tissue–Derived Exosomes as a Novel Therapeutic Approach for Refractory Alopecia Areata: A Report of Two Cases

**DOI:** 10.1155/crdm/4908438

**Published:** 2025-10-14

**Authors:** Mohammad Ali Nilforoushzadeh, Nazila Heidari, Amirhossein Heidari, Niloufar Najar Nobari

**Affiliations:** ^1^Skin Repair Research Center, Jordan Dermatology and Hair Transplantation Center, Shahid Beheshti University of Medical Sciences, Tehran, Iran; ^2^Skin and Stem Cell Research Center, Tehran University of Medical Sciences, Tehran, Iran; ^3^School of Medicine, Iran University of Medical Sciences, Tehran, Iran; ^4^Faculty of Medicine, Tehran Medical Sciences, Islamic Azad University, Tehran, Iran

**Keywords:** adipose tissue, alopecia areata, exosome, treatment

## Abstract

Alopecia areata (AA) is an autoimmune condition with unpredictable progression, negatively impacting the quality of life. Despite various therapeutic options, refractory cases remain challenging to treat. Emerging evidence highlights the regenerative and immunomodulatory potential of exosomes, particularly in tissue repair and hair follicle regeneration. Herein, we aim to evaluate the therapeutic efficacy of adipose tissue–derived exosomes (ASC-exosomes) in two patients with severe, refractory AA. Two female patients with chronic AA resistant to multiple conventional treatments underwent a single session of intradermal administration of exosomes derived from adipose tissue. Baseline evaluations, including the Severity of Alopecia Tool (SALT) score, were conducted and reassessed 3 months posttreatment. Clinical and laboratory parameters were monitored throughout the follow-up. Both patients demonstrated significant hair regrowth and improvement in SALT scores (Case 1: 79.6–59.6; Case 2: 70.2–58.6) without adverse events. The treatment was well-tolerated, with sustained clinical benefits observed alongside adjunctive topical minoxidil. ASC-exosomes represent a promising and safe therapeutic strategy for refractory AA, combining immunomodulatory and regenerative properties. Further randomized, controlled studies are necessary to validate these findings and refine cell-free therapeutic protocols for broader clinical applications.

## 1. Introduction

Alopecia areata (AA) is a persistent autoimmune, non-scarring condition characterized by immune system attacks on hair follicles, nails, and sometimes the retinal pigment epithelium [1]. AA can be presented as an acute self-resolving disease over a period of 6–12 months or as a chronic condition with relapsing and remitting courses [2]. This disorder specifically targets anagen-phase hair follicles, resulting in hair loss while preserving the potential for follicle regeneration. The lifetime incidence rate of this condition is about 2.1%, affecting people of all ages with an equal distribution between males and females [3, 4]. Based on the distribution pattern, various forms of AA include the following: patchy AA, which is the most common form; alopecia totalis; alopecia universalis; alopecia incognita or diffuse AA; ophiasis; and sisaipho [1]. Within 20 years, disease recurrence will occur in all AA cases [5]. The sudden onset, complex pathophysiology, and unpredictable course of AA negatively influence the quality of life in affected cases [6].

The management of AA involves both hair regrowth and preservation of hair regrowth [7]; however, spontaneous remission is common in cases of AA, ranging from 68% in patients with less than 25% scalp involvement to 8% in those with more than 50% scalp involvement [8]. In cases of failed spontaneous remission and for those who are anxious about their condition, a broad range of treatment options is applicable based on the disease severity and the subject's age [7]. These treatments vary from topical medications, such as topical and intralesional corticosteroids, to systemic treatment with novel immune-modulating agents, including Janus kinase (JAK) inhibitors [1].

Exosomes, which are small extracellular vesicles, hold promising potential in the field of regenerative medicine, including acute cardiovascular events, atopic dermatitis, neurodegenerative disorders, and motor neuron injury [9]. Exosomes can stimulate receptor cells and enhance tissue repair through transporting different substances, such as proteins, RNA, and microRNA [10]. Furthermore, exosomes offer several advantages over mesenchymal stem cells (SCs) (MSCs), including ease of storage and transportation, enhanced biosafety, and reduced immunogenicity. These findings underscore the potential of exosomes in managing alopecia conditions. Herein, we describe two AA cases successfully treated with adipose tissue–derived exosomes (ASC-exosomes).

## 2. Case Presentation

### 2.1. Case 1

**Figure 1:**
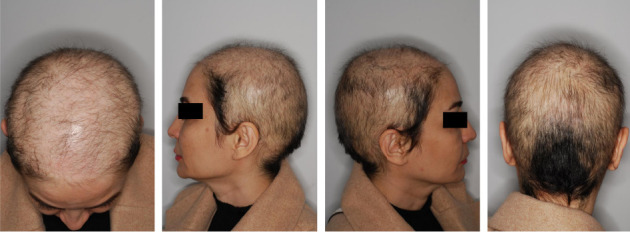
Pretreatment scalp condition of Case 1, showing significant hair loss in the frontal, lateral, and occipital regions.

A 43-year-old female patient exhibited a 20-year history of severe sisaipho-Type AA, accompanied by significant psychological distress. At the time of admission, the initial severity of the disease was 79.6 based on the Severity of Alopecia Tool (SALT) score evaluation. A generalized hair loss with no remarkable inflammation was noticed across the frontal, temporal, and parietal scalp but spared the occipital region (Figure 1). She did not mention a positive family history of AA. Her medical history was otherwise unremarkable for systemic diseases, apart from the significant emotional burden associated with AA. Before admission to this center, the patient visited various dermatologists and underwent different therapeutic approaches, including intralesional and oral steroids, oral tofacitinib, platelet-rich plasma (PRP), cyclosporin, and azathioprine; however, she failed all previous treatments, and her condition got worse over time.

### 2.2. Case 2

**Figure 2:**
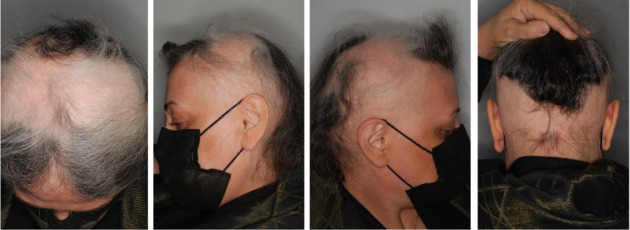
Pretreatment scalp condition of Case 2, highlighting extensive hair loss in the frontal, lateral, and occipital areas.

A 63-year-old female patient presented with a 2-year history of severe AA. Upon admission, the initial severity of the disease was evaluated using the SALT score, which was reported as 70.2. Physical examination revealed hair loss with no apparent inflammation over the vertex in the temporal, parietal, and occipital areas (Figure 2). Also, she reported a negative family history of AA. Furthermore, her medical history did not depict any significant systemic diseases. Notably, the patient previously failed various medications, including oral and topical steroids, azathioprine, and baricitinib.

### 2.3. Intervention Method

Before commencing treatment with human-derived exosomes, the patients underwent a thorough laboratory assessment, which included a complete blood count with differential, a comprehensive metabolic panel, a fasting lipid profile, and screening for HIV, tuberculosis, and hepatitis B and C. Moreover, an informed consent form was obtained from both patient subjects during the treatment initiation. The method of exosome harvesting from AT has been described in the previous investigation [11]. The exosome was administered in one session, containing 2 mL of the exosome. The targeted area was prepared 30 min before the procedure by cleansing with sterile gauze and decontaminating with 70% alcohol pads. After that, local anesthesia was induced by injecting a 2% lidocaine solution to establish ring-block anesthesia. The procedure was subsequently performed by applying intradermal injections to the targeted areas of the scalp using a 1-cc syringe fitted with 27-gauge needles. The injection sites were spaced approximately 0.5–1 cm apart. The patient was then recommended to avoid scalp cleansing for the following 24 h.

### 2.4. Follow-Up

**Figure 3:**
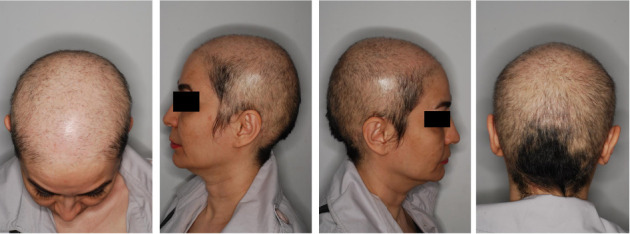
Posttreatment scalp condition of Case 1 at the 3-month follow-up, demonstrating noticeable hair regrowth and increased density across all regions.

**Figure 4:**
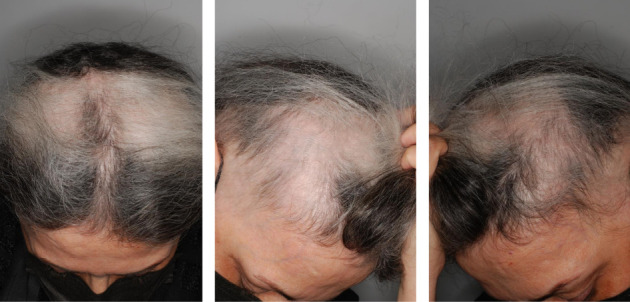
Posttreatment scalp condition of Case 2 at the 3-month follow-up, showing visible improvement with reduced hair loss and hair regrowth in the affected areas.

Three months following the exosome injection, both cases were visited again. Signs and symptoms of severe AA were remarkably improved at the time of the visit (Case 1: SALT score: 59.6; Case 2: SALT score: 58.6) with significant hair restoration as well as alopecia resolution. In addition, the laboratory results remained within normal limits at the 3-month follow-up examination. Figures 3 and 4 illustrate the patient's (Cases 1 and 2, respectively) SALT score and clinical status at the 3-month follow-up. Both patients are now receiving topical minoxidil 5% and are scheduled for re-evaluation every 2 months to monitor the progress of treatment.

## 3. Discussion

In this study, ASC-exosomes were investigated as a treatment for AA in two cases that failed their previous medications. Both cases underwent a single treatment session, and the outcomes were clinically promising and satisfactory for both patients and the treatment team.

The exact pathophysiology of AA is still unclear; however, it has been suggested that this disease arises from an autoimmune reaction to the hair follicles following genetic or environmental triggers [12]. To be more precise, in the AA, hair follicles and dendritic cells display autoantigens to the CD8+ T-cells, CD4+ T-cells, and natural killer (NK) cells by promoting the activity of major histocompatibility complex (MHC) Class I and MHC Class II [13]. Moreover, hair follicle cells secrete cytokines such as interferons, which recruit more inflammatory cells and stimulate inflammatory secretion, and cascades such as the JAK-signal transducer and activator of transcription pathway in the CD8+ T-cells, consequently resulting in a positive loop development [12]. Ultimately, these events interfere with the hair growth cycle, causing AA development, which results in negative psychological consequences, such as anxiety and depression in affected cases [14].

Treatment of AA presents significant challenges and yields a wide range of outcomes. Corticosteroids are the most common treatment utilized for AA treatment; however, they carry the risk of cutaneous atrophy at the site of application [15]. PRP is another treatment option acting through prolongation of the anagen phase and inserting antiapoptotic effects on dermal papilla cells [16], demonstrating variable results in clinical studies [17, 18]. Novel immunomodulatory agents, such as JAK inhibitors, offer promising results as alternative treatment options [12]. Nonetheless, disease exacerbation often occurs following the cessation of these treatments. Moreover, the impact of these therapeutic options on the final natural course of the disease is unpredictable, highlighting the unmet medical need for patients with recurrent and extended AA [7].

Recent evidence-based studies in regenerative medicine have confirmed that utilizing a variety of related therapeutic methods is safe and efficacious in restoring normal function to diseased tissues or organs, including hair follicles [19, 20]. SCs such as MSCs have the potential to promote the regeneration of hair follicles and skin components, as well as to generate new hair follicles through organoid technology [21]. Bone marrow–derived MSC transplantation in a murine model of AA has shown promising results by blocking NK group 2D (NKG2D) receptors, which are involved in a signaling pathway that contributes to the pathogenesis of AA [22]. Cell-free products, such as extracellular vesicles, exosomes, and conditioned medium, also play a crucial role in modifying the hair follicle cycle and regeneration [23]. Utilization of MSC-derived extracellular vesicles in a murine alopecia model revealed an increase in dermal papilla proliferation and migration [24]. Moreover, subcutaneous injection of MSC-derived extracellular enriched vesicles in a case of persistent chemotherapy-induced hair loss resulted in a dramatic improvement with complete regrowth of terminal hair [25].

ASC-exosomes have gained attention as a novel therapeutic strategy for AA due to their regenerative potential and ability to modulate follicular biology. Experimental data indicate that ASC-exosomes enhance the proliferation of human dermal papilla cells (hDPCs) and increase the expression of key hair growth–associated genes, including alkaline phosphatase (ALP), versican (VCAN), β-catenin, and lymphoid enhancer–binding factor 1 (LEF-1), largely via activation of the Wnt/β-catenin signaling pathway [26]. These molecular effects are accompanied by stimulation of hair shaft elongation and increased ALP activity, suggesting their capacity to promote follicular regeneration. Another preclinical study on a murine model of AA illustrated that human umbilical MSC-derived exosomes hold promise in regulating the hair growth cycle by enhancing hair follicle keratinocyte proliferation and migration [10]. Although the precise mechanism of action remains unclear, it is speculated that exosomes facilitate crosstalk between epithelial and MSCs during the hair growth cycle by regulating paracrine signaling [27]. Moreover, it has been found that dendritic cell–derived exosomes modulate immune responses by interacting with immune cells and regulating antigen-presenting cell and T-cell activity by MHC Class I and Class II [28]. Clinical studies also in androgenetic alopecia (AGA) have reported that ASC-exosome therapy, particularly when combined with microneedling, can significantly improve hair density, thickness, and overall clinical appearance over 12–24 weeks, with high patient-reported satisfaction and no serious adverse effects [29, 30]. These findings highlight the potential of exosomes as a therapeutic option for AA, possessing both immunomodulatory and hair regrowth properties. However, further investigations are warranted to substantiate these results and to assist specialists in selecting the most effective medical practice.

This case report has several limitations, including a small sample size, the absence of a control group, and reliance on subjective outcome measures. Objective evaluations, such as trichoscopy or biomarker analysis, were not performed, and the relatively short follow-up reduces the ability to assess long-term efficacy. In addition, the concurrent use of minoxidil introduced a potential confounding factor, and standardized protocols for exosome preparation and administration were not applied. These limitations underscore the need for future clinical studies with larger cohorts, objective assessments, longer follow-up periods, and appropriate control groups to better establish the therapeutic role of ASC-derived exosomes in AA.

The current study showed the effectiveness of AA treatment with ASC-exosomes in two cases. Further research involving a larger number of patients and different MSC sources, such as fat and placenta, will provide more insight into the efficacy of cell-free therapies in AA.

## Data Availability

The data that support the findings of this study are available from the corresponding author upon reasonable request.
